# Plasmablastic lymphoma in the ano-rectal junction presenting in an immunocompetent man: a case report

**DOI:** 10.1186/1752-1947-5-168

**Published:** 2011-05-03

**Authors:** Mayur Brahmania, Thomas Sylwesterowic, Heather Leitch

**Affiliations:** 1 Department of Medicine, Division of Gastroenterology & Hematology, St Paul's Hospital, Vancouver, BC, V5Z 1M9, Canada

## Abstract

**Introduction:**

Plasmablastic lymphoma is an aggressive non-Hodgkin lymphoma classically occurring in individuals infected with HIV. Plasmablastic lymphoma has a predilection for the oral cavity and jaw. However, recent case reports have shown lymphoma in the stomach, lung, nasal cavity, cervical lymph nodes and jejunum in HIV-negative individuals. We report what is, to the best of our knowledge, the first case of plasmablastic lymphoma occurring in the ano-rectal junction of an HIV-negative man.

**Case Presentation:**

A previously healthy 59-year-old Caucasian man presented with painless rectal bleeding. Colonoscopy revealed a lesion in the ano-rectal junction, with pathological examination demonstrating atypical lymphoid cells consisting primarily of plasmablasts with rounded nuclei, coarse chromatin, small nucleoli and multiple mitotic figures. Immunohistochemical analysis showed the atypical cells were negative for CD45, CD20, CD79a and immunoglobulin light chains, but were strongly positive for CD138 and EBV-encoded RNA. The results were consistent with a diagnosis of plasmablastic lymphoma. Aggressive systemic chemotherapy and involved field radiation therapy resulted in complete clinical and pathological remission.

**Conclusion:**

Increasing awareness of plasmablastic lymphoma in HIV-negative individuals and in this location is warranted.

## Introduction

Plasmablastic lymphoma (PBL) is most frequently an AIDS-related non-Hodgkin lymphoma (NHL) and is usually confined to the oral cavity and jaws, although involvement of distant sites may occur [1-6]. It is a rapidly progressive tumor usually seen in human immunodeficiency virus (HIV) infection with advanced immunodeficiency (CD4<200 cells/ml) and, like NHL, is an AIDS defining illness [7,8]. In recent years, cases of PBL have been reported involving the lungs [9], stomach [10], cervical lymph nodes [11], nasal cavity [12] and jejunum [13] in HIV-negative individuals. We report the first case of PBL to be found in the ano-rectum of an HIV- negative man.

## Case presentation

A 59-year-old heterosexual Caucasian man presented with recurrent and profuse rectal bleeding. Past medical history was remarkable for an ischiorectal abscess, with no apparent predisposing conditions, which was incised and drained. Eventually our patient had developed an anal fistula which was managed with Tisseel^® ^(a surgical adhesive composed from fibrinogen and thrombin). Later a seton, a length of suture material looped through a fistula to keep it open and allow pus to drain, was inserted. The seton was exchanged and tightened on three occasions and eventually was extruded. Physical examination at that time showed no remaining fistula. Our patient was investigated with a gastrointestinal series and colonoscopy which were negative for inflammatory bowel disease and malignancy.

At lymphoma presentation, the history was otherwise unremarkable; in particular, there was no history of noticeable lumps, unexplained fevers, drenching sweats, or weight loss. There were no symptoms related to cytopenia. General physical examination was unremarkable, with no palpable lymphadenopathy or hepatosplenomegaly. Digital rectal examination showed scarring of his right peri-anal area and a small, tender, ulcerated mass was palpable in his anal canal at the nine o'clock lithotomy position. There was no blood on the examining glove. Laboratory investigations showed his complete blood count (CBC), electrolytes, liver panel, calcium, and lactate dehydrogenase levels to be within normal range. A serum protein electrophoresis showed no monoclonal protein; however there was a slight decrease in the gamma fraction at 8 g/L (lower limit of normal 10 g/L). A screen for hepatitis B and C was negative, as was serology for varicella zoster virus, Epstein-Barr virus (EBV), cytomegalovirus, herpes simplex virus and HIV.

**Figure 1:**
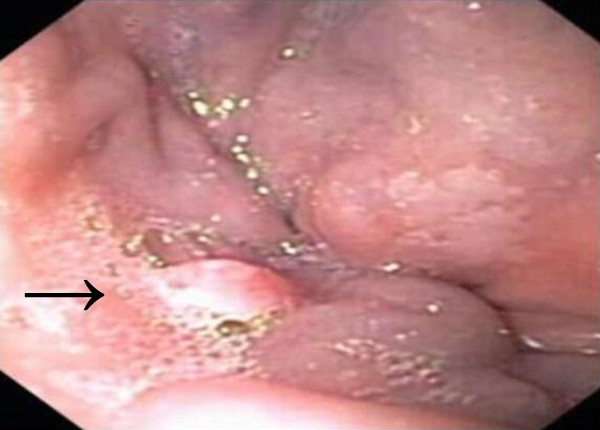
**Mass at the ano-rectal junction**.

**Figure 2:**
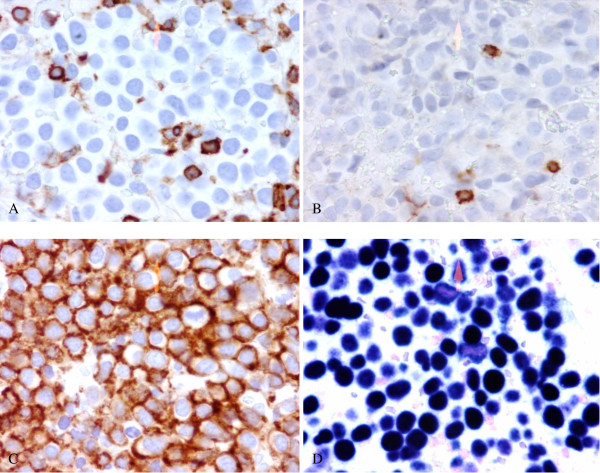
**Immunohistochemical staining (a) CD45 (b) CD20 (c) CD138 (d) EBER**.

Our patient underwent a colonoscopy which showed a normal colon apart from a 5 mm polyp at 20 cm which was hyperplastic by pathologic examination. At the ano-rectal junction, a hypervascular cauliflower-like mass of 3 mm was seen and biopsied (Figure 1). Histopathological examination demonstrated abundant atypical large lymphoid cells with lesser numbers of plasma cells. The atypical lymphoid cell population consisted predominantly of plasmablasts with rounded nuclei, coarse chromatin, small nucleoli and multiple mitotic figures. Immunohistochemical analysis showed the atypical cells were negative for CD3, CD5, CD10, CD20, CD30, CD45, CD56, BCL-2, BCL-6, CD45 (Figure 2a), CD20 (Figure 2b), CD79a. Furthermore, we could not detect any restriction of immunoglobulin light chains (kappa or lambda), or expression of immunoglobulin heavy chains IgG, IgM, IgD; however there was cytoplasmic expression of IgA. In contrast, the neoplastic cells were strongly positive for MUM1, epithelial membrane antigen, CD38, CD138 (Figure 2c) and EBV-encoded RNA (EBER) (Figure 2d). There was no expression of LANA-1. The proliferation index by Ki-67 immunohistochemistry was approximately 70%. The results were consistent with a diagnosis of PBL.

Staging investigations included a computed tomography (CT) scan of the chest, abdomen and pelvis, which showed no evidence of lymphoma in these other sites. A bone marrow aspirate and biopsy was negative for lymphoma. Our patient was staged as Ann Arbor 1A (Additional file 1: Table S1), and was low risk according to the International Prognostic Index. Our patient subsequently underwent gallium scanning, which showed increased activity in his right inguinal region (2 cm), suggestive of gallium avid lymphoma.

Our patient was treated with three cycles of CHOP chemotherapy (cyclophosphamide, doxorubicin, vincristine and prednisolone), in full doses and on schedule, followed by involved field radiation therapy to the ano-rectal region, pelvic nodes, and right inguinal nodes. The chemotherapeutic regimen and radiation therapy were well tolerated by our patient and no complications were reported. A CT scan done following therapy showed complete resolution of previously detected abnormalities. CT scanning at six months from lymphoma diagnosis showed no evidence of recurrence. Most recent clinical follow up was done five years from diagnosis with rectal examination and colonoscopy showing ongoing remission.

## Discussion

PBL is usually diagnosed in the context of HIV infection, however in recent years it has also been reported in a number of sites in HIV-negative individuals [9-13]. As seen from our case report, it can also be found in the hindgut. Derived from B-cells, PBL has distinct morphologic and immunophenotypic features by which it has been defined [14,15]. PBL has some morphologic characteristics similar to diffuse large B-cell lymphoma (DLBCL) and the World Health Organization classifies PBL as a variant of DLBCL. However, PBL is differentiated from DLBCL by minimal or no expression of CD20 and leukocyte common antigen. Instead, PBL has been characterized by the plasmablastic morphology of the neoplastic cells, with numerous mitotic figures, the expression of plasma cell markers such as VS38c and CD138/syndecan-1 [1,3,15] and EBER positivity [16].

PBL has been shown to have an immunophenotype and tumor suppressor gene expression profile virtually identical to that of the plasmablastic variant of plasma cell myeloma. In contrast, this profile is unlike that of DLBCL, suggesting a cell of origin more in keeping with myeloma than NHL. However, unlike myeloma, and unlike the majority of DLBCL in immunocompetent individuals, it was found that most HIV-positive patients with PBL were EBER-positive [16].

Evidence supporting a pathogenic role for human herpes-virus-8/Kaposi's sarcoma-associated herpes virus (HHV-8/KSHV) in promoting lymphoma cell growth has been described almost exclusively in HIV-related cases of PBL and/or multicentric Castleman's disease [17-20]. In these disorders, an interaction between HIV and HHV-8 has been suggested, whereby viral interleukin-6 may provide a mitogenic stimulus resulting in enhanced proliferation of HIV in patients co-infected with both viruses, in addition to supporting the survival of infected lymphocytes, thus predisposing them to transforming events [21-25]. Our HIV-negative patient had no evidence of infection by HHV-8.

It is unclear if PBL is associated with a relative state of immunosuppression in HIV-negative patients. Although our patient was HIV negative, it is possible the recurrent problems with abscess formation and fistulas may have led to a state of relative immunosuppression and development of lymphoma, or the ongoing inflammation may have promoted survival of lymphocytes which then underwent further transforming events. Alternatively, the recurrent abscesses may have been secondary to a previously unrecognized state of relative immunosuppression, as indicated by the decrease in gamma globulins demonstrated on serum protein electrophoresis. In a case series reported by Teruya-Feldstein *et al*. [26], two out of six cases of PBL in HIV-negative individuals occurred in the setting of iatrogenic immunosuppression; one was a recipient of a renal allograft with localization of PBL to the skin of the leg [27] and the other a patient with ulcerative colitis receiving azathioprine [28]. Both cases were EBV positive. It has been documented that EBV-positive Hodgkin lymphoma may be associated with Crohn's disease [29,30], providing further suggestion that immune dysregulation may play a role in the development of PBL. While a minority of HIV-negative patients have EBV-positive NHL, EBV positivity is more frequently associated with immunosuppression-related lymphoma, and the EBV positivity of the PBL in our patient further supports that he may have had a state of relative immunosuppression.

Current guidelines for the treatment of lymphoma in early stage include CHOP or similar chemotherapy regimens, with or without involved field radiation therapy. In the case studies of HIV-negative individuals with PBL, all including our patient received CHOP. Future therapies may take into account the infection of lymphoma cells with EBV and possibly HHV-8, and the similarities of these cells to plasma cells, and may direct therapy toward these specific features.

## Conclusion

We report a case of a patient with PBL, an aggressive NHL usually associated with significant and documented immunosuppression, which can occur in immunocompetent individuals, most usually in the gastrointestinal tract. Biopsy, with accurate pathological and immunohistological testing is essential for the correct diagnosis and planning subsequent therapy.

## Consent

This report was prepared in accordance with requirements of the Institutional Research Ethics Board. Written informed consent was obtained from the patient for publication of this case report and any accompanying images. A copy of the written consent is available for review by the Editor-in-Chief of this journal.

## Competing interests

The authors declare that they have no competing interests.

## Authors' contributions

MB conceptualized, designed and was a major contributor in writing the manuscript. TS performed the colonoscopy. HL was a major contributor in writing the manuscript. All authors read and approved the final manuscript.

## Supplementary Material

Additional file 1 **S1: Ann Arbor staging classification for Hodgkin and Non-Hodgkin lymphomas**. The table shows the different stages of both Hodgkin's and Non-Hodgkin's lyphomas. Click here for file
