# Supplementary material for: Plasmablastic lymphoma in the ano-rectal junction presenting in an immunocompetent man: a case report
Source: J Med Case Rep. 2011 May 3;5:168. doi: 10.1186/1752-1947-5-168 (PMC3107799; doi:10.1186/1752-1947-5-168)
Supplement: Additional file 1 — S1: Ann Arbor staging classification for Hodgkin and Non-Hodgkin lymphomas. The table shows the different stages of both Hodgkin's and Non-Hodgkin's lyphomas. [file 1752-1947-5-168-S1.DOC]

Legend 1. Ann Arbor staging classification for Hodgkin and Non-Hodgkin Lymphomas

| Stage I | Involvement of a single lymph node region (I) or of a single extralymphatic organ or site (IE) |
| --- | --- |
| Stage II | Involvement of two or more lymph node regions or lymphatic structures on the same side of the diaphragm alone (II) or with involvement of limited, contiguous extralymphatic organ or tissue (IIE) |
| Stage III | Involvement of lymph node regions on both sides of the diaphragm (III) which may include the spleen (IIIS) or limited, contiguous extralymphatic organ or site (IIIE) or both (IIIES) |
| Stage IV | Diffuse or disseminated foci of involvement of one or more extralymphatic organs or tissues, with or without associated lymphatic involvement |

All cases are subclassified to indicate the absence (A) or presence (B) of the systemic ("B") symptoms of significant unexplained fever, night sweats, or unexplained weight loss exceeding 10 percent of body weight during the six months prior to diagnosis.

Adapted from Carbone, PP, et al, Cancer Res 1971; 31:1860 and Lister, TA, et al, J Clin Oncol 1989; 7:1630.
